# Strengthening institutions for public health education: results of an SWOT analysis from India to inform global best practices

**DOI:** 10.1186/s12960-022-00714-3

**Published:** 2022-02-19

**Authors:** Emily Miller, Megha Reddy, Preetika Banerjee, Haley Brahmbhatt, Piyusha Majumdar, D. K. Mangal, Shiv Dutt Gupta, Sanjay Zodpey, Anita Shet, Meike Schleiff

**Affiliations:** 1 Department of International Health, Johns Hopkins Bloomberg School of Public, 415 N Washington Street 5th Floor, Baltimore, MD 21223 United States of America; 2 Indian Institute of Health Management Research, Jaipur, Rajasthan India; 3 Public Health Foundation, India (PHFI), Delhi, India

**Keywords:** Public health, Education, Training, Capacity strengthening, Healthcare workforce, Human resources, India

## Abstract

**Background:**

Developing public health educational programs that provide workers prepared to adequately respond to health system challenges is an historical dilemma. In India, the focus on public health education has been mounting in recent years. The COVID-19 pandemic is a harbinger of the increasing complexities surrounding public health challenges and the overdue need to progress public health education around the world. This paper aims to explore strengths and challenges of public health educational institutions in India, and elucidate unique opportunities to emerge as a global leader in reform.

**Methods:**

To capture the landscape of public health training in India, we initiated a web-based desk review of available offerings and categorized by key descriptors and program qualities. We then undertook a series of in-depth interviews with representatives from a purposively sample of institutions and performed a qualitative SWOT analysis.

**Results:**

We found that public health education exists in many formats in India. Although Master of Public Health (MPH) and similar programs are still the most common type of public health training outside of community medicine programs, other postgraduate pathways exist including diplomas, PhDs, certificates and executive trainings. The strengths of public health education institutions include research capacities, financial accessibility, and innovation, yet there is a need to improve collaborations and harmonize training with well-defined career pathways. Growing attention to the sector, improved technologies and community engagement all hold exciting potential for public health education, while externally held misconceptions can threaten institutional efficacy and potential.

**Conclusions:**

The timely need for and attention to public health education in India present a critical juncture for meaningful reform. India may also be well-situated to contextualize and scale the types of trainings needed to address complex challenges and serve as a model for other countries and the world.

## Introduction

Historically, educational systems that produce the healthcare workforce in countries of all income levels have been inefficient in preparing adequate numbers or appropriate skill mixes of workers needed to solve complex health system challenges [1]. This holds true in India, with problems compounded by understaffed health systems, workforce shortages, under-subscribed training programs and inadequate distribution of qualified workers [2, 3]. The significance of public health education in India has gained considerable traction in recognition of current and projected human resource gaps that threaten progress towards global health priorities. This has led to further exploration of the public health training landscape, providing insights needed to develop a robust public health workforce and calls to ensure that public health education takes priority to combat anticipated needs [2–5].

Even defining what constitutes as public health education can present challenges, as a wide range of disciplines ranging from clinical programs to healthcare administration to community medicine are often encompassed within the public health training ecosystem due to overlap of topics areas or historical connections. There have been recent efforts to standardize public health education, including the development of the Ministry of Health and Family Welfare Model Curriculum Handbook for Master of Public Health (MPH) [6], which aimed “*to prepare competent cadre of professionals who have a basic understanding of the various aspects of public health and are able to successfully apply this knowledge towards meeting public health challenges in Indian Context.”*

The number of public health trainings and institutions offering them in India has increased, although demand within both public and private sectors for trained personnel remains low [2]. Students graduating with qualifications such as Master of Public Health (MPH) degrees have faced uncertain career pathways– despite demonstrated public health needs.

Equipping a strong public health workforce requires not only an increase of trained professionals but focus on the quality of training [4]. Similar to other countries, public health in India has traditionally been addressed through a medicalized lens [7, 8] lacking appropriate collaboration with other disciplines. As a result, a multidisciplinary workforce to contextualize health more broadly beyond the largely biomedical approach as run out of medical schools is needed. In addition, public health training and community medicine in India have largely been considered a backup for medical students compared to paths in medicine considered more prestigious. This leads to reduced participation and underinvestment in these training programs, further exacerbated by a lack of clear career opportunities for those with such qualifications [7].

To better understand some of these complexities surrounding public health education in India, we undertook a landscape analysis of various public health training institutions, followed by further collection and analysis of perceptions from some of their affiliates. By analyzing the relationships and patterns between internal and external factors across institutions, in this paper we aim to illustrate how experts at leading institutions in India perceive cross-institutional and larger ecosystem challenges to identify opportunities towards a more interconnected and optimized approach to public health training.

## Methods

We initiated a landscape analysis to explore contributors that influence public health training in India. First, we conducted a desk review of institutions offering public health education in India. The training programs were catalogued by institution by capturing a set of key descriptors of the training program(s) offered, including geographical location, types of qualification(s) offered, core competencies, etc.

Based on the desk review, we then utilized a qualitative research approach and strategic management orientation. We drew on aspects of constructivism and pragmatism to both build on and learn from previous study and focus on the application of our data into practice, respectively. We conducted a series of in-depth interviews with a purposive sample of key informants from institutions identified in the desk review. Last, we conducted an overall analysis of Strengths, Weaknesses, Opportunities and Threats (SWOT) from themes identified across interviews which were reflective of the larger educational environment in which institutions are operating.

### Desk review

We employed a web-based search to identify public health training offerings across India using specific search terms (Appendix 1). A variety of different types of training programs were identified (e.g., diplomas, postgraduate, doctoral). To go beyond individual care and observe a macro-level lens of training, this study focused on programs where training involved broader aspects of public health and not specialized community medicine programs with a more clinical focus [9, 10], and thus we excluded the latter from the desk review.

### In-depth interviews

A series of 13 in-depth interviews were conducted with faculty and other representatives from a purposive sample of institutions identified from the desk review. Interview guides were developed based on previous competency-based education literature [11] to capture data regarding institutional departments, core research areas, courses offered, mentorship models followed, and collaborations. We conducted 1-h interviews over a 3-month period between September and December 2020 by three data collectors who were trained to administer in-depth interviews. Owing to the COVID-19 pandemic, interviews were conducted remotely over Zoom. Privacy was maintained throughout the process of data collection. Participant consent was obtained prior to initiating and recording each interview. Data collectors conducted interviews using a semi-structured interview guide and maintained notes during the process.

We conducted interviews until we achieved data saturation on key study themes. We aimed to capture diverse experiences of institutions across India from our sampling frame of institutions identified through the desk review. We did not include all 59 institutions due to practical considerations and since, after data saturation is achieved, further interviews do not yield sufficient new information to warrant burden on respondents. De-identified audio records of the interviews were retained in a secure database accessible to the research staff and transcribed by an established transcription service.

Transcribed interviews were uploaded into Dedoose (version 8.0.35, Los Angeles, CA, USA) qualitative data analysis software for thematic analysis. Transcripts were reviewed individually by the research team members, using a data-driven approach to identify key themes and develop potential parent codes and associated child codes with descriptors included as examples. We then held a workshop to finalize the code book by collating, defining, and refining the codes based on consensus. The final codebook consisted of 10 parent codes and 32 child codes. Analysis was conducted by a team of four, with each transcript coded by an initial reviewer followed by a secondary review by a different study team member to ensure consistency of coding across interviews. Weekly team calls were conducted to establish consensus on data interpretation, organize quotes and understand themes emerging across transcripts.

### SWOT analysis

SWOT analysis aims to appraise internal and external factors and can be used for the purpose of strategic planning [12]. Internal factors were identified based on whether they are inherent to or within the control of the institution, such as faculty or enrollment, while external factors pertained to larger environmental influences that might include political, social, or economic factors. Using key themes from the qualitative analysis we plotted the SWOT analysis chart based on whether they were internal, external, positive, or negative. Finally, we categorized the leading themes in each SWOT category into larger domains.

## Results

### Overview of public health degree programs

**Fig. 1:**
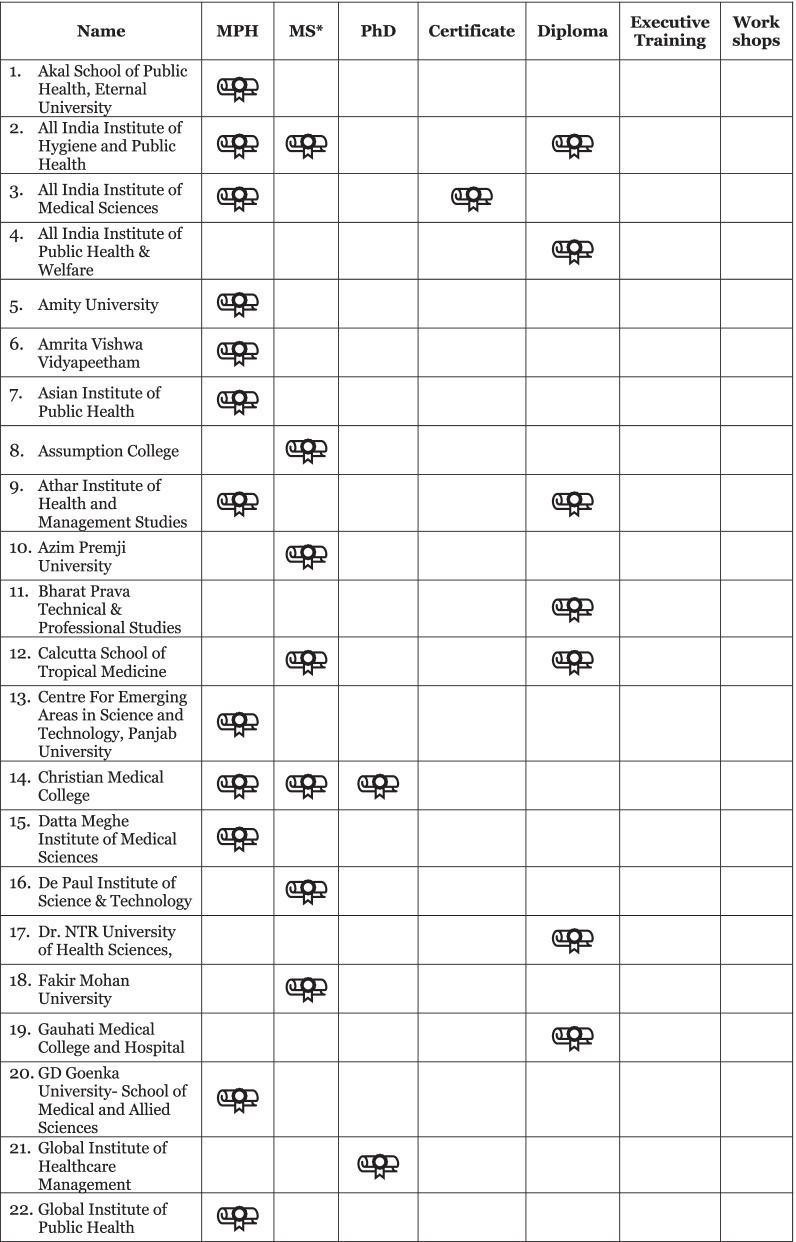

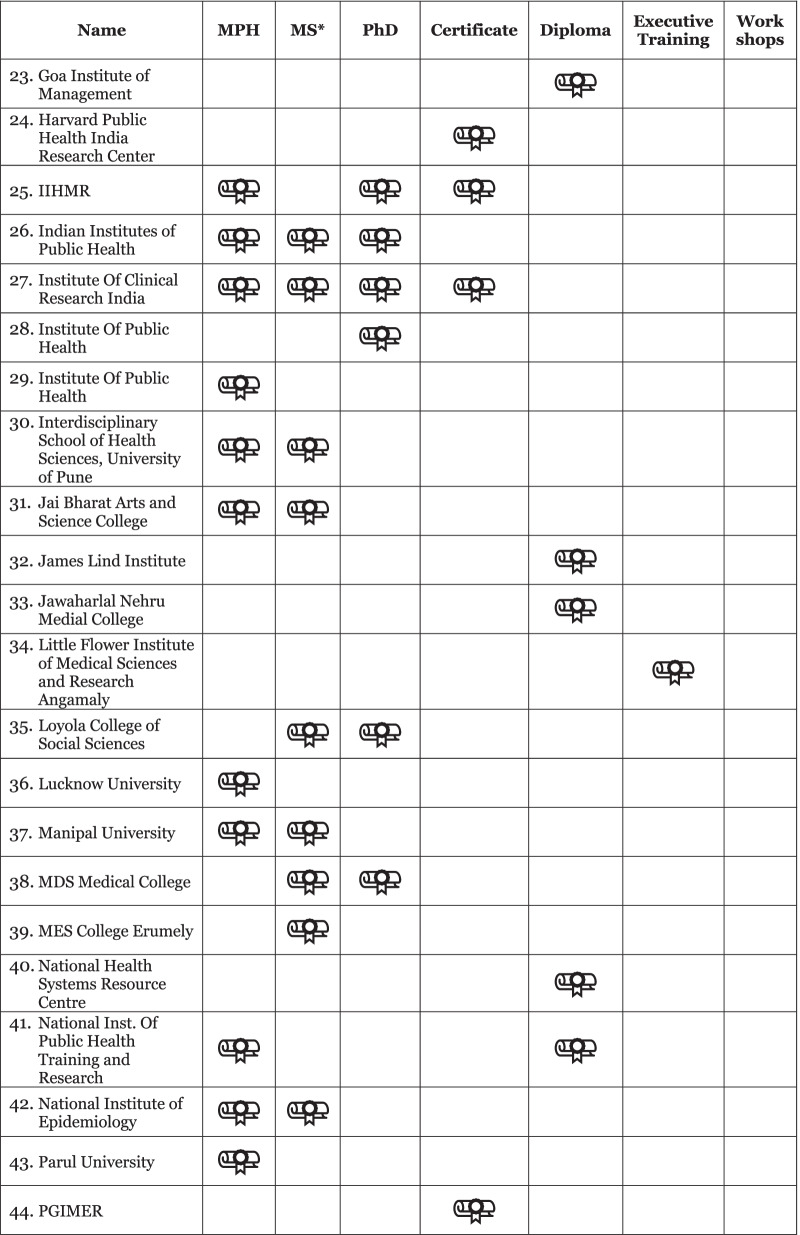

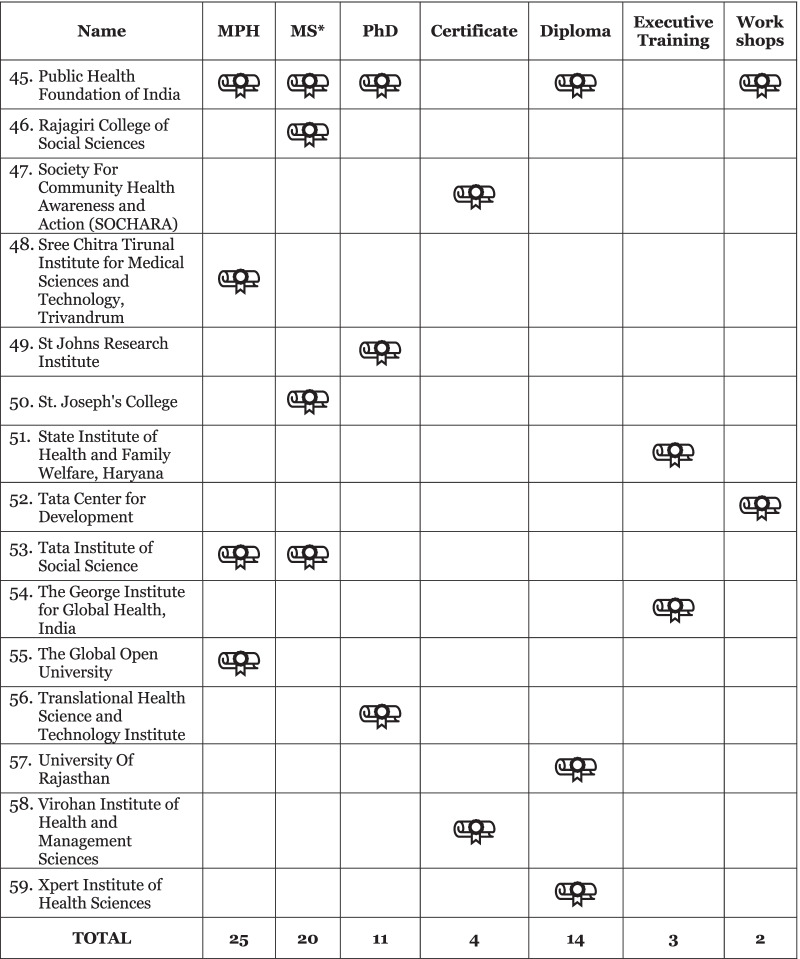
Public health education programs available in India based on desk review. *Master of Science (MSc) programs were identified from related disciplines and applications relevant to public health practice, including Epidemiology, Population Studies, Disaster Management, Health Informatics and Applied Nutrition

Our initial data set from the desk review, supplemented by discussion with key stakeholders in India and literature review, yielded a heterogenous collection of 59 institutions across many universities, medical colleges, colleges of social sciences, and institutes of research and technology. Of these 59 institutions identified, the breakdown of programs included 25 Master of Public Health (MPH); 20 Master of Science; 14 diplomas; 11 PhDs; four certificates; three executive trainings and two workshops (Fig. 1). This included representation from both private and government institutions (54% and 55% of identified institutions, respectively), public private partnerships, as well as training hubs outside of traditional academia, including a community health resource center. Geographically, 41 cities across 21 states were represented, and three institutions had branches in multiple locales.

### SWOT analysis results

**Fig. 2:**
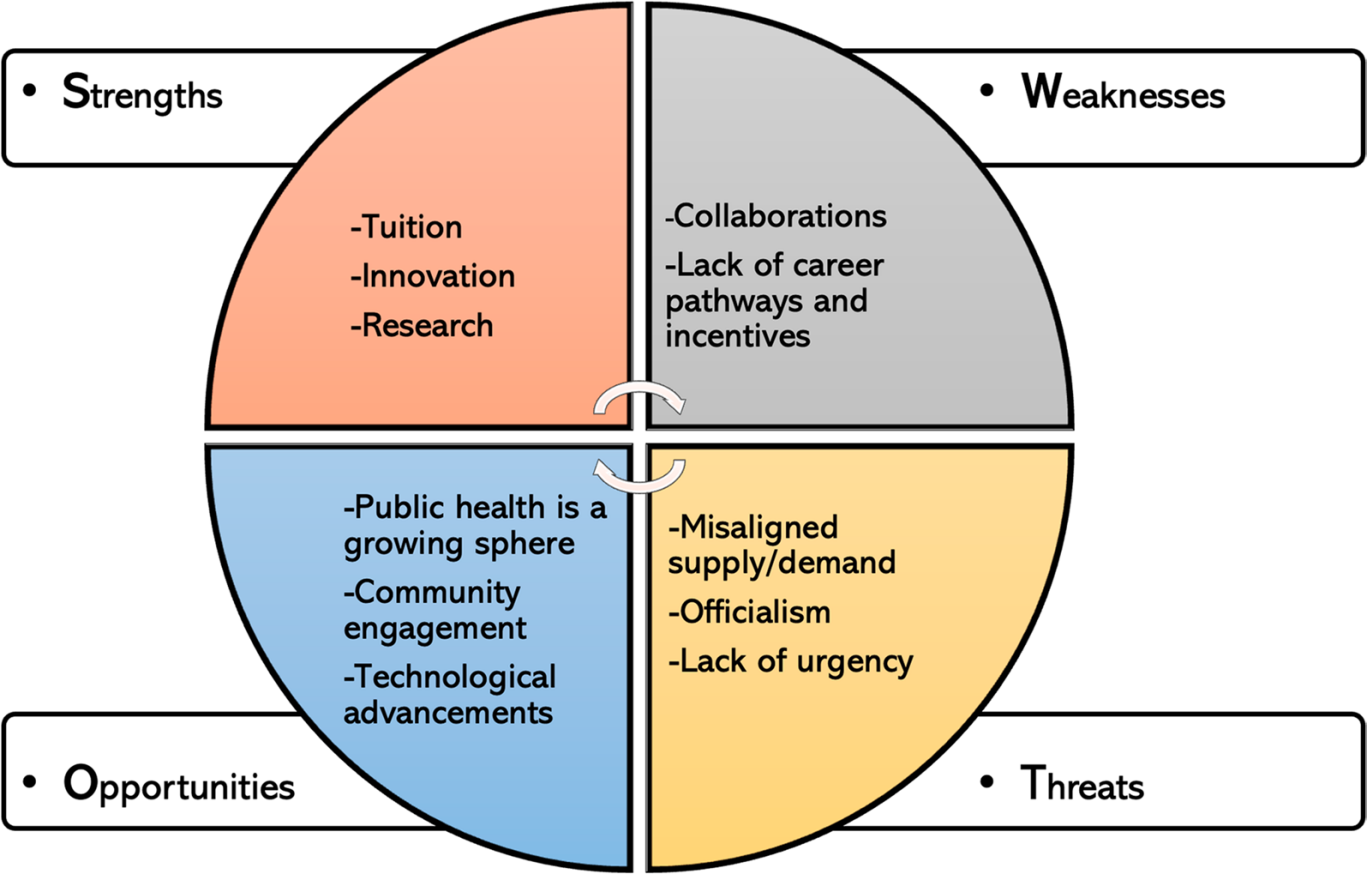
SWOT analysis

Given the variability of the institutions identified and interviewed, the strengths and weaknesses (internal to institutions) and opportunities and threats (external to institutions) identified were not universally represented, but rather emerged as common factors that can enhance or hinder successful public health educational programs and institutions (Fig. 2). Themes could be potentially identified as a strength or a weakness depending on the institution itself, and as such we have presented our findings as guidance for institutions and stakeholders interested in considerations for future work or identifying solutions to challenges rather than broad characterizations of the institutions.

### Key strengths: tuition, innovation, research

*Tuition* Affordable tuition, particularly at institutions with public funding sources, was recognized as an enabling factor for building public health educational capacity. Tuition fees at such institutions were described in terms such as “*nominal”* and considered financially accessible. Representatives from government universities often juxtaposed the affordability of their own institutions against private universities. Yet this is not necessarily a universal perception, as captured by one representative at a private university:

*Our fee structure…[is] higher…compared to all other universities or institutions or schools offering similar program in the country. But still, we are able to attract people. Usually, our stake is almost 100% full every time and the reason for this is our reputation. (2021.02.16.DK.HB)*

In addition, many respondents identified a variety of student support opportunities available, including scholarships, stipends, sponsorship for professional development opportunities such as conferences, as well as support reserved for traditionally underrepresented communities such as scheduled castes/scheduled tribes (SC/STs).

*Innovation* Intrinsic innovation, or innovation from faculty members and institutional leaders, emerged as a key strength of institutions across the board. This was particularly evident in the context of COVID-19. One interviewee recounted, *“the faculty were very significantly involved in directly advising government on a real-time basis, developing protocols, being part of advisory committees…developing guidelines…” (128ASPB).*

COVID-19 also required institutions to adapt their learning and training programs, which was generally perceived to have been successful. Multiple institutions already had some level of virtual trainings available that were able to be quickly scaled, and distance learning was described by one respondent as a well-known modality that had been offered since 1991. Faculty have been successful in teaching courses in an online format, summarized by one interviewee, “*We ensured that there is no disruption in the teaching… clearly, we could learn the new ways of continuing our activities. And we were able to do it well.” (216DKHB).*

*Research* One respondent directly stated that*, “Our strength is the focus of our research…” (216DKHB)* Throughout many of our interviews, research capacities and high research productivity were identified as institutional strengths. Several respondents mentioned having their institutional origins rooted in research. Considerable faculty investment, prominent collaborations, in-house expertise and multidisciplinary faculty bodies were some of the cited examples of how these institutions lead and disseminate evidence-based contributions to public health and its related disciplines to India and beyond. High expectations for developing student research competencies were also commonplace.

### Key weaknesses: collaborations, lack of career pathways and incentives

*Collaborations* While inter- and cross-institutional partnerships and collaborations were the norm and highly valued, many respondents did not feel as though they were always being utilized to their full potential. There was a perceived need to better align common goals, improve working relationships, and structure interactions. One government university interviewee shared that while networks were established, there existed untapped potential to fully bring out strengths:

*There are a lot of things we can learn from each other, the typical rights-based NGOs, typical corporations for profit, typical government institutions... there is a huge opportunity of learning from each other, and I think there is much scope to gain from experiences from each other. That is one thing we would like to improve. (1127CASMR)*

The need for mutually conceived and owned partnerships was also noted, described by one interviewee as, “*collaborations on equal footing…the design, the ideation, all of this is there is co-ownership, that is something which is much more appreciated” (16266-17865).*

*Lack of career pathways and incentives* A perceived lack of motivating factors for public health professionals to pursue further qualifications was frequently mentioned, including encouraging career opportunities or lack of prestige. One interviewee further noted that trainings were not necessarily met with professional advancement, noting, *“Even if you get additional degree [related to public health], in many states it really doesn’t matter…Yes, knowledge wise or skill wise, it will be useful, but not career wise.” (1209CASMR).*

Lack of lucrative or at least dependable compensation, unappealing work conditions and other challenges for graduates were also raised. Remote or rural areas, for example, can be challenging to create desirable career opportunities for trainees. It was also mentioned that mentors for emerging professionals (e.g., non-faculty) often could not receive any financial compensation in such roles, straining human capital.

### Key opportunities: interest in public health, community engagement, technological advancements

*Public health is a growing sphere* The role of COVID-19 in garnering public health awareness and interest was a consistent theme. One interviewee observed that, *“public health came to the fore*” as the pandemic has continued *(042246MCHI)*. It was mentioned on two separate occasions that, since COVID-19 emerged, suddenly everyone seemed to know what an epidemiologist does. A marked increase in applicants for their programs, since the pandemic began was oft noted, and growth of research activities was also cited by several interviewees, despite investigations being disrupted by COVID-related restrictions.

While evident that the reach of public health is expanding, exactly how it will evolve is less clear, as one interviewee reflected:

*COVID has given us an opportunity to work with governments and to get governments to see the advances of working with academic institutions, and I think it could be really interesting to see how many of these opportunities turn into long-term relationships, post-COVID. (128ASPB)*

*Community engagement* Several institutions had community engagement opportunities built into their education and research initiatives, noting unique opportunities to present learners with real-world challenges and cultivate soft skills, such as empathy and adaptability. These collaborations were highly regarded where present. In addition, bringing in community voices can inform public health priorities, as described by one interviewee:

*I think our greatest strength is that we’re in touch with communities…we noticed there are [often] gaps between what the public health system thinks or what public health as a discipline accepts to what’s actually happening on the ground. (2020.10.15CASMR)*

*Technological advancements* Many respondents felt as though technological adaptations borne from COVID-19 restrictions brought new opportunities for public health education. In the case of research activities, for example, one respondent noted, *“one thing that has opened up is this whole thing of online research…using Google Forms, using telephonic interviews, using Zoom…this is going to stay.” (12ASPB)* Employing digital platforms also brings potential to improve and extend collaboration and communication. Mentees and mentors, researchers and other stakeholders can convene virtually to leverage expertise and foster innovation without the constraints of geographic limitations or hassle of travelling long distances.

### Key threats: misaligned supply and demand, officialism, lack of urgency

*Misaligned supply and demand* Considerable emphasis was placed on the need to harmonize the supply and demand factors of public health training*.* The demand for individuals with public health qualifications is lacking, as one respondent explained, “*There is no recognition of this discipline [public health] as a separate discipline and there are no jobs in the government sector clearly requiring the public health qualifications.”(216DKHB)* Without clear career pathways, public health trainings remain undersubscribed, whereas an increase in posts could heighten demand.

The geographic distribution of public health trainees presents an additional challenge, as some states observe an abundance or “*clustering*” of qualified trainees, while other states lag, even with concerted advocacy efforts.

It was also recognized that improving the supply and demand for public health training must be designed with consideration to health system needs. As summarized by one interviewee:

*Since the past 15 years or so, there has been a growth in public health education in the country…but I think there’s a need for…much greater collaboration between the public health educational institutions…a systematic way of trying to understand what are the human resource needs. (1015CASMR)*

*Officialism* It was mentioned that sometimes bureaucratic processes designed to oversee academic programs could result in overly rigid systems at the institutional level. Factors such as quotas for faculty posts or curriculum mandates that were not necessarily current were sometimes met with frustration or perceived to limit autonomy. The challenges of balancing these types of mandates with actual needs was further described:

*So, actually the [recommended] combination or the proportion of medical faculty is quite skewed. Now, we have less medical faculty and slightly more non-medical faculty, but the actual posts are, you know, the same that they’re supposed to be. (1207SGHB)*

*Lack of urgency* Many interviewees mentioned insufficient capacity to prepare for public health challenges despite the pending reality of more severe and frequent major public health events. The inevitability of “the next pandemic” was cited often, as were the increasing complexities of public health challenges (e.g., dual burden of disease, rising inequalities, emerging/re-emerging infectious diseases, epidemiological and demographical transitions). One interviewee summarized the scenario:

*I hope our policymakers are beginning to realize that these kinds of episodes [like pandemics] are going to get more and more frequent, and also there’s so many other things involved in this. There’s climate change, there’s environmental degradation, there’s urbanization... And, unless we have a really strong primary care network, we’re going to… have to face this kind of a crisis. (202010.20CASMR)*

It emerged that strengthening public health response will require not only scaling up of the public health workforce but a shift into how they are prepared, phrased by one respondent as “*building*” public health education from a more interdisciplinary and broader framework.

## Discussion

These insights captured foster mutual learning and joint solutions related to public health education, which can help create an ecosystem to share knowledge, human resources and technologies across institutions and borders [1]. Through this landscape and SWOT analysis, we found that the scope of public health education and training in India is expansive and multifaceted. Public health training institutions recognize their robust research culture, affordability to students, and reputation for innovation. There is a felt need to optimize collaborations and harmonize training-employment tracks. Opportunities to harness momentum surrounding public health and technological advances should be seized, as should effective strategies to better involve communities. Misconstructions surrounding the needs and roles of public health education should be further explored to navigate obstacles impacting institutional efficacy and potential.

Public health education institutions were perceived as financially accessible and nimble, and these enabling factors position India to elevate public health education and reach high cohorts of learners in an environment where such skills are highly needed. These strengths, coupled with strong research capabilities, make India an ideal leader to scale training reform and bolster the public health workforce to deliver evidence-based, population-focused solutions to complex real-world challenges.

The value of collaborations in public health education was recognized, as was the potential to improve them. The complexities of health education benefit from networks between and within training institutions, government ministries, health sector employers, and other stakeholders [13]. That said, partnerships can be challenging to manage, and there can be difficulties associated with how to effectively share expertise or navigate competing priorities or power dynamics. Regional networks such as the Asia Pacific Network on Health Professional Education Reform (ANHER), The Southeast Asian Public Health Educational Institutional Network (SEAPHEIN) and India Public Health Education Institutions Network (India PHEIN) exist and should be leveraged and cultivated to better promote knowledge exchange, share problems and solutions regarding human resources for health, and encourage south–south collaboration [3].

There was concern surrounding the lack of clear professional paths and incentives for public health professionals. The creation of public health-specific posts and standardizing pathways to career advancement are a natural fit to increase demand for public health education [14]. There is also potential to explore motivating factors to attract and retain talent, particularly in areas difficult to staff. Access to context-specific, high-quality training, for example, can incentivize and motivate health professionals [15].

The experts we spoke with noted a tangible upturn of interest in public health. Still in the throes of a catastrophic pandemic, the time is ripe to leverage attention towards public health to improve and expand public health education in India and beyond. Recent lessons present an opportunity to build back better and develop locally responsive, population-centered health systems. Public health education should involve trainees with opportunities to build relationships with local governments and health centers and interact with community health workers (CHWs) and social leaders. These activities enable public health professionals to better understand community needs and withstand local difficulties [16].

Constraints in India’s public health workforce [4] lend an opportunity to focus on both supply and demand-driven factors of workforce development. A forecast of supply-need gaps in requirements for public health professionals in India showed that in the absence of feasible interventions, the country is likely to fall short of the required public health professionals by nearly 45,000 by the year 2026 [5]. Expansion should be intentional and with consideration of health system needs and gaps. Institutions can contextualize trainings to the needs of the populations they serve [17] and prepare learners with problem-solving capabilities needed for everyday scenarios [7]. In addition, having a national accreditation system that formally recognizes public health education and its curriculum across institutions can help ensure quality and bring recognized value.

Advocacy and policy can play a role in aligning supply and demand—particularly while momentum is high because of COVID-19—to build prestige and recognize the merit of public health as its own discipline. Findings from models that have worked well in other settings could be useful to inform these efforts. The United States and Canada, for example, have benefited from concerted efforts to evolve their public health workforce, achieved through design and implementation of strategies to optimize workforce size, composition, training, competencies, recruitment, effectiveness, and retention [18, 19]. Opportunities to target policy measures towards health workforce gaps in India have been recently identified [20]. These include expanding training institutions across underrepresented geographies, honing technologies that improve service delivery and training outcomes, prioritizing skills–trainings matched to identified workforce gaps, and the development of robust, integrated data systems efficiently capture human resource for health capacities.

Solutions for public health challenges are multifaceted and require a holistic, systems-oriented understanding of health. Other studies exploring the shortcomings of public health education suggest that competency-based education frameworks are not consistently employed, and curricula are sometimes built on dated pedagogies that fall short to prepare for real-world contexts [1, 3, 4]. Globally, planetary health and One Health approaches are largely underutilized in public health education, which prioritize primary health, overall wellbeing over curative services, and address rising threats from emerging zoonotic disease and climate change [21, 22]. Embracing these frameworks can play a key role in capacitating the next generation of public health leaders. Although there remains work ahead, India has substantial potential to align education with sustainable development and global health priorities [23].

While our findings yielded helpful lessons, they are not without limitations. Using an Internet-based search as the primary mode for the desk review meant we were unable to capture institutions without an online presence. We also did not capture medical public health education nor any educational institutions in languages besides English. Furthermore, the information available on different institution websites was highly variable, presenting a challenge to extract uniform data for every institution identified. While key interviews presented a rich perspective across a variety of institutions, the ability to generalize these findings across all public health education training institutions in India is limited. Despite these recognized limitations, we gained an improved understanding as to how public health education institutions are successful and offer encouraging insight for opportunities that remain.

## Conclusion

The need to prioritize institutional capacity for public health education in India has escalated from urgent to critical. India is hardly alone in this sense, yet equipped with a unique combination of need and potential, could serve as a global model for intentional, rapid reform. The COVID-19 pandemic has served to reemphasize the importance of public health education and identify important lessons. The values of transdisciplinary knowledge-sharing, cross-sectoral collaborations and reciprocal partnerships for public health training are recognized, but there is more to learn about optimization. The future of public health training must be responsive to the evolving, interdependent, real-world environments in which health outcomes are shaped, and thus institutions should embrace fluidity and challenge conventional paradigms.

## Data Availability

All data from the desk review is available online and is compiled and presented in the figures in the paper. Deidentified interview transcripts are available upon reasonable request from the corresponding author.

## Appendix 1: Search Terms

Google “India” AND “public health” OR “master’s in public health” OR “community health” OR “health diploma” OR “population health” OR “public health school” OR “public health institution” OR “global health institute” OR “public health training” OR “community health training” OR “public health curriculum” OR “public health student”

## Abbreviations

ANHER: Asia Pacific Network on Health Professional Education Reform
CHW: Community health worker
MPH: Master of public health
MSc: Master of science
NGO: Non-governmental Organization
PHEIN: India Public Health Education Institutions Network
SC/ST: Scheduled castes scheduled tribes
SEAPHEIN: The Southeast Asian Public Health Educational Institutional Network
SWOT: Strengths weaknesses opportunities threats
